# Smart biocomposite hydrogels in action: unraveling the roles of lignin, temperature, and crosslinker on drug release

**DOI:** 10.1039/d5ra05933j

**Published:** 2025-11-07

**Authors:** Missoury Wolff, Eric M. Davis

**Affiliations:** a Department of Chemical and Biomolecular Engineering, Clemson University Clemson SC 29631 USA ericd@clemson.edu

## Abstract

In this study, the release kinetics of a model water-soluble drug, caffeine, from thermoresponsive biocomposites, comprised of lignin, poly(*N*-isopropylacrylamide) (PNIPAm), and poly(vinyl alcohol) (PVA) were studied. Specifically, two series of soft biocomposites were fabricated—one containing softwood Kraft lignin at a 2 : 2 : 1 (lignin : PNIPAm : PVA) mass ratio and one at a 2 : 3 (PNIPAm : PVA) mass ratio, with the latter serving as a control membrane to those containing lignin. The crosslink density of these soft biocomposites was altered by systematically varying the concentrations of both glutaraldehyde, the crosslinker for lignin and PVA, and methylenebisacrylamide, the crosslinker for PNIPAm, between 5 and 15 mass%, respective to the dry polymer masses. At room temperature, the introduction of lignin in the membranes led to a reduction in diffusivity. Notably, the diffusivity of caffeine from membranes with 5 mass% crosslinker was seen to decrease by approximately two orders of magnitude when compared to the control membranes. However, in lignin-containing composites synthesized with 15 mass% crosslinker, caffeine diffusivity increased by nearly an order of magnitude at temperatures above the volume phase transition temperature of PNIPAm compared to the same membrane at room temperature. The most significant increase occurred in the highest concentration studied. Across the membranes studied, the diffusivity of caffeine did not exhibit any consistent trends with varying crosslinker composition. In addition to caffeine release kinetics, the equilibrium water uptake (EWU) of each membrane was measured. In general, the EWU was seen to decrease with increases in crosslinker concentrations.

## Abstract 2

Lignin-containing, thermoresponsive hydrogels demonstrated improved sustained caffeine release, eliminating the initial burst effect commonly observed in conventional hydrogels.

## Graphical abstract

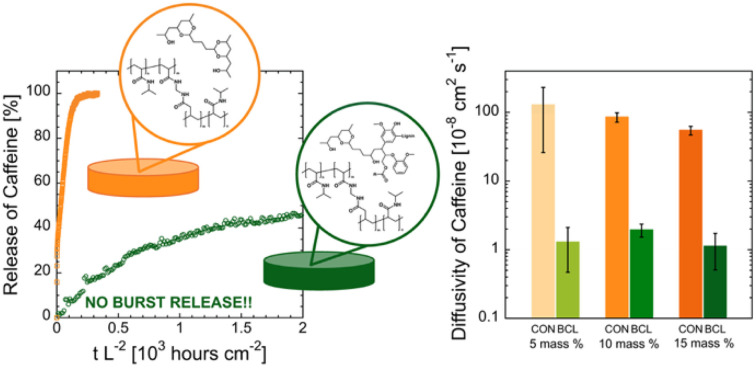

## 1. Introduction

A hydrogel is a viscoelastic material composed of crosslinked polymer chains forming a three-dimensional network with the capacity to absorb and retain substantial quantities of water, often exceeding 1000% of its dry mass.^1–3^ Their hydrophilic nature makes them particularly valuable in drug delivery systems, where both absorption and release of pharmaceutical compounds can be finely tuned through manipulation of the network structure.^4,5^ To enhance control over drug release, a myriad of research has focused on stimuli-responsive materials—*i.e.*, materials whose network structure changes in response to environmental triggers such as temperature, pH, light, and electrical charge.^6–8^ Among these, the thermoresponsive polymer poly(*N*-isopropylacrylamide) (PNIPAm) is the most widely studied. PNIPAm exhibits reversible water solubility and undergoes a coil-to-globule transition when heated above its lower critical solution temperature (LCST) of approximately 32 °C.^9^

When chemically crosslinked into a three-dimensional network, thermoresponsive polymers exhibit a discontinuous change in volume at a characteristic temperature known as the volume phase transition temperature (VPTT).^10^ Conventional drug delivery systems utilizing PNIPAm leverage its sol–gel transition above the LCST, whereby a physically crosslinked hydrogel forms upon heating.^11^ In intravenous applications, PNIPAm is first loaded with bioactive agents in its sol state and subsequently injected into the body. As the temperature rises from ambient to physiological levels, the polymer undergoes gelation, entrapping the drug within the forming hydrogel network for controlled release.^12^

However, this method is limited by a significant drawback—the phase transition is not instantaneous. During the gelation process, a portion of the drug is released prior to full network formation, leading to what is known as the “burst release”.^13^ This premature release can reduce therapeutic efficacy, as the body's metabolic processes may be unable to absorb or utilize the drug efficiently at such elevated concentrations. The result is not only diminished clinical benefit but also economic and environmental inefficiencies, as excess drug is excreted and may ultimately enter wastewater systems.^14^ Since PNIPAm-based hydrogels are intended to improve dosing frequency and consistency relative to conventional administration routes, this uncontrolled initial release undermines the primary advantage of the system.

To mitigate burst release and extend the effective duration of drug action, interpenetrating polymer networks (IPNs) have been explored. These systems promote mesh-controlled diffusion, offering enhanced regulation over drug transport kinetics by physically restricting premature diffusion through a more stable and tightly structured network.^15,16^ The use of IPNs allows for the incorporation of multiple polymers, with various properties of interest, to create composite materials with properties that are advantageous for each application. Notably, there are several limitations to using PNIPAm by itself, such as poor mechanical strength and limited water permeability.^17,18^ To combat these issues, other hydrophilic polymers like poly(vinyl alcohol) (PVA) have been incorporated to form IPNs with PNIPAm, resulting in enhanced water flux and mechanical robustness.^19–21^ For example, researchers have leveraged PNIPAm–PVA hydrogels for the controlled release of the cancer therapeutic Doxorubicin^22^ and antifungal drug Voriconazole,^23^ where the concentration of PVA was altered to tune the rate of drug release.

However, polymers like PNIPAm and PVA are synthesized from petroleum-derived feedstocks, perpetuating our dependence on non-renewable resources. Consequently, the integration of biopolymers as sustainable alternatives in hydrogel systems has gained significant attention.^24–29^ One such candidate is lignin, an abundant byproduct of paper and pulp industry, which offers a renewable source of hydroxyl-rich functionality. In addition to its sustainability, lignin is inherently biocompatible and exhibits antimicrobial and antioxidant properties,^30–32^ making it a promising component for biomedical applications.^33^

In this study, thermoresponsive, biocomposite hydrogels, consisting of lignin, PNIPAm, and PVA, were fabricated at a mass ratio of 2 : 2 : 1 lignin : PNIPAm : PVA. Control membranes—thermoresponsive, composite hydrogels without lignin—were also fabricated, at a mass ratio of 2 : 3 PNIPAm : PVA. To better understand the impact of crosslink density (that is, the network structure) on drug release kinetics of these biocomposites, the crosslinker concentrations of both glutaraldehyde (GA), the crosslinker for lignin and PVA, and methylenebisacrylamide (MBA), the crosslinker for PNIPAm, were varied between 5 and 15 mass%. The release kinetics of a model hydrophilic drug, caffeine, were measured for all membranes, which were loaded with approximately 12 mg of caffeine per gram of polymer mass. Using an early-time solution to Fick's 2nd law, the diffusivity of caffeine from the soft biocomposites was calculated from the release kinetics at both room temperature and 40 °C. In addition to drug release studies, the equilibrium water uptake of each hydrogel was measured. Finally, electron microscopy was used to image the hydrated network structure for each membrane at both room temperature and 40 °C.

## 2. Experimental section

### 2.1. Reagents and materials

Dimethyl sulfoxide (DMSO) (ACS reagent, ≥99.9%), ammonium persulfate (ACS Reagent, ≥98.0%), methylenebisacrylamide (MBA) (99%), *N*,*N*,*N*,*N*′-tetramethylethylenediamine (TMEDA) (ReagentPlus®, 99%), and glutaraldehyde (GA) solution (50 mass% in water) were purchased from Sigma-Aldrich. *N*-Isopropylacrylamide (NIPAm) was purchased from Tokyo Chemical Industry. Methylene blue (MB) was purchased from VWR Analytical. Poly(vinyl alcohol) (PVA) (MW = 78 000 g mol^−1^, 98% hydrolyzed) was purchased from Polysciences, Inc. Crude-bulk softwood Kraft lignin (weight average molecular weight (*M*_W_) ≈ 16 800 g mol^−1^, ash content ≈ 0.89 wt%, referred to as BioChoice™ lignin) was obtained from Domtar (Fort Hill, South Carolina, USA). Caffeine was purchased from Ward's Science.

### 2.2. Hydrogel synthesis

First, NIPAm was dissolved in DMSO in a round bottom flask to create a 45% mass/mass (m/m) solution. Separately, in a 20 mL vial, lignin was added to DMSO until a 10% m/m solution was achieved, and the mixture was sonicated (using an ultrasonic sonicating bath) until dissolved (typically 1–3 hours). In another round bottom flask, the 78 k MW PVA was incrementally added to DMSO heated to 90 °C until the 5% m/m solution was transparent and homogeneous. The polymer solutions were mixed at a 2 : 2 : 1 NIPAm : lignin : PVA ratio for the BCL membranes and at a 2 : 3 NIPAm : PVA ratio for the control membranes, and high-purity nitrogen gas was bubbled through for two hours to sparge the solution.

Separately, a small round bottom flask of DMSO was sparged for approximately 30 minutes. In a 20 mL vial, ammonium persulfate (APS) was dissolved in the sparged DMSO and deionized water in a mass ratio of approximately 1 : 4 : 5 APS : water : DMSO to create a solution containing 30 mass percent APS of the NIPAm mass. The APS solution was then sonicated until dissolved. Next, in another 20 mL vial, methylenebisacrylamide (MBA) was dissolved in the sparged DMSO to create a 10 mass% solution. The amount of MBA in the vial varied between 5, 10, and 15 mass% of NIPAm. This vial was also sonicated until all solids were dissolved. Lastly, caffeine was dissolved in the remaining sparged DMSO at a concentration of 2.9 mg mL^−1^ of sparged DMSO. Note that this concentration was chosen as it is below the reported solubility value of caffeine in DMSO, which is 3 mg mL^−1^.

Once the round bottom flask containing the polymers and DMSO was sparged for two hours, the MBA solution was added. Following five minutes of stirring, the round bottom flask was moved to an inert atmosphere maintained by a glovebox, where the glutaraldehyde solution (50 mass% in water) was added. Following immediate removal from the glovebox, the solution was again stirred for five minutes. Next, the APS solution was added to the mixture and the standard five minutes of stirring occurred. Then, *N*,*N*,*N*,*N*′-tetramethylethylenediamine at a concentration of 5 mass percent of the total masses of NIPAm, MBA, and APS was added to the solution. After an additional five minutes of stirring, the drug solution was added to the mixture.

Once the solution had stirred for a final five-minute interval, the solution was cast into polytetrafluoroethylene dishes. The membrane was then moved into an oven heated to 60 °C, and a partial vacuum was pulled. The vacuum was increased incrementally, with occasional short periods of release so that the moisture could be wiped from the oven walls, until most of the DMSO was removed from the film. Once the majority of the DMSO was removed, the hydrogels were placed under full vacuum for 12 hours, resulting in a robust, free-standing hydrogel. Next, membranes were left on the benchtop until visibly dried (approximately 7–10 days). When it was time to measure the caffeine diffusivity from each film, the membrane was placed under dynamic vacuum for at least two hours.

### 2.3. Caffeine release kinetics and diffusivity

**Fig. 1:**
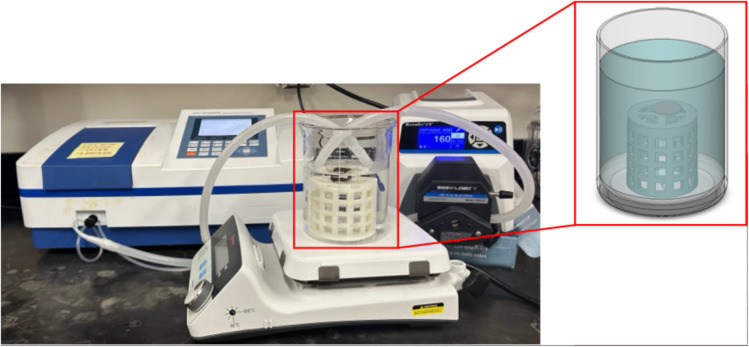
Image of experimental set up for continuous caffeine release studies using the UV-vis spectrometer and flow-through cuvette.

To capture caffeine release from the drug-loaded membranes, an in-house drug release apparatus was created. Shown in Fig. 1 (boxed in red), a polymer cage was 3D printed (Lulzbot Mini 2; Fargo, North Dakota), which served to elevate the membrane above the stir bar, preventing any unintentional agitation of the membrane that might affect the release of caffeine. Within a large beaker, the 3D-printed cage was attached with polydimethylsiloxane (PDMS). In this case, the PDMS acts to ‘glue’ the cage to the glass beaker once cured and crosslinked. The container was filled with approximately 500 mL of deionized water, and a background measurement was taken after stirring for about ten minutes. This volume was selected to fully submerge the membrane while minimizing dilution, which could reduce UV-vis absorbance and lower the signal-to-noise ratio. The beaker was then placed on a heating plate, with the water temperature maintained either at room temperature or at 40 °C. At *t* = 0, a dry, caffeine-loaded membrane was placed on top of the cage, and the stir bar was set to approximately 350 rotations per minute to ensure the solution was thoroughly mixed to maintain the perfect sink condition.

Data collection was taken in one of two ways: manual or automatic collection. For the manual collection, small aliquots were removed from the container at different time intervals, and the caffeine concentration was measured *via* ultraviolet-visible light (UV-vis) spectroscopy (VWR, UV-3100PV), scanning from a wavelength of 300 nm to 200 nm. We note that the peak of interest for caffeine is centered around 274 nm.^34^ Immediately following measurement, the aliquot was returned to the beaker to maintain a constant volume. For automatic collection (shown in Fig. 1), a peristaltic pump (Masterflex L/S® with Easy-Load® II Pump Head) was used to continuously flow the aqueous solution between the container and a flow-through UV-vis cuvette (FireflySci). For this approach, samples were continuously taken every 30 or 60 seconds, depending on the release rate of the caffeine.

Using a calibration curve, the measured absorbance from the UV-vis spectrometer can be equated to the mass of caffeine in solution over time. From these data, the diffusivity of the drug of interest from the polymer membrane can be calculated using the following equation^35^1
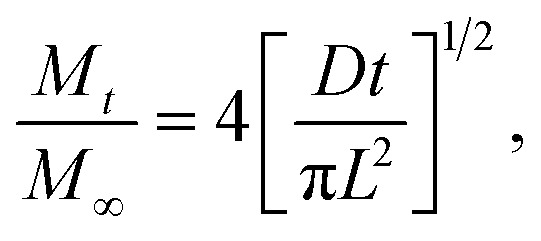
where *M*_*t*_ and *M*_∞_ are the mass of drug released at time *t* and as time approaches infinity, *t* is the time elapsed, *L* is the thickness of the membrane, and *D* is the diffusivity of the drug from the membrane and is the only fitting parameter in the equation. In using this equation, the following assumptions were made: (1) the surface concentration is kept such that it maintains the perfect sink condition and (2) the primary route of diffusion is in the direction of thickness. We note that this equation only applies to the “early-time release data”, or up to 60% of the drug released by mass. The diffusivity coefficient of caffeine for each membrane was obtained from the least sum of squares analysis between the experimental data and model.

### 2.4. Equilibrium water uptake (EWU)

The equilibrium water uptake of each hydrogel was determined at both room temperature (RT, ∼21 °C) and above the volume phase transition temperature at 40 °C. Membranes were rinsed in deionized water for at least 24 hours with three water exchanges to ensure no drug remained in the hydrogel. Afterward, each hydrated membrane was gently tapped with a Kimwipe™ to remove excess water, and the mass of the hydrogel was measured *via* an analytical lab balance (Mettler Toledo ME204E). The membrane was then transferred into a 20 mL vial and placed in a warm water bath set to 40 °C for at least 6 hours. Again, a Kimwipe™ was used to dab off the excess water from the surface of the membrane and the mass at the elevated temperature was recorded. Each membrane was then dried at 50 °C under dynamic vacuum for 24 h. Finally, the hydrogels were removed from the oven, and the dry hydrogel mass was recorded. The equilibrium water uptake was calculated by using the following equation2
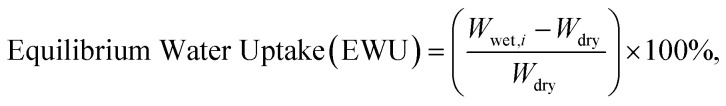
where *W*_wet,*i*_ is the hydrated mass at *i* = room temperature or 40 °C and *W*_dry_ is the dry mass of each membrane.

### 2.5. Characterization of hydrogel network structure *via* scanning electron microscopy (SEM)

The network structure of the swollen hydrogels was characterized using a variable-pressure scanning electron microscope system (Hitachi SU-5000). Prior to preparing the samples for imaging, the membranes were hydrated in DI water for at least 24 hours. In the case of the hydrogels assessed at 40 °C, hydrogels were heated to 40 °C for at least 4 hours. Next, the swollen hydrogels were lyophilized. The hydrogels were freeze-dried for 24 hours at −105 °C and 0.100 mbar (using a Labconco FreeZone freeze dryer). Prior to inserting samples into the SEM, the samples were attached to an aluminum specimen holder by conductive carbon adhesive tape and sputter-coated with a thin layer of platinum (approximately 30 Å) using an Anatech Ltd Hummer 6.2 sputtering system. SEM imaging was performed under high pressure with an accelerating velocity of 120 kV, a spot intensity of 30 nanoamperes, and a working distance of 10 mm.

## 3. Results and discussion

### 3.1. Biocomposite hydrogel synthesis

The nomenclature used to describe the hydrogels throughout this manuscript is detailed in Table 1. For example, a lignin-containing hydrogel with 10 mass% crosslinker is denoted as “BCL-10XL”, while those soft composites containing no lignin – *i.e.*, the control (CON) membrane – is denoted as “CON-10XL”.

**Table 1:** Nomenclature for lignin : PNIPAm : PVA biocomposite hydrogels

	Lignin : PNIPAm : PVA mass ratio [—]	Crosslinker concentration [mass%]	Nomenclature [—]
Control (CON)	0 : 2 : 3	5	CON-5XL
		10	CON-10XL
		15	CON-15XL
BioChoice™^,^^a^ lignin (BCL)	2 : 2 : 1	5	BCL-5XL
		10	BCL-10XL
		15	BCL-15XL

a BioChoice™ is the trademarked name of the Kraft lignin obtained from Domtar.

**Fig. 2:**
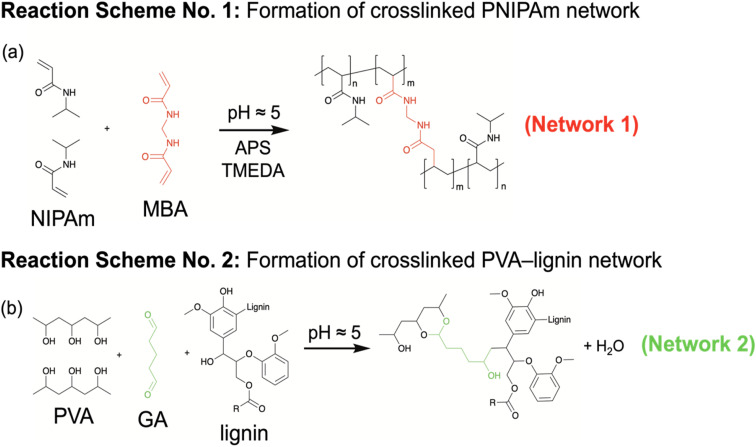
Reaction schematics of the two distinct interpenetrating networks, (a) PNIPAm and (b) PVA + lignin, formed within the hydrogels during membrane fabrication.

Furthermore, illustrative schematics of the two reaction schemes for the fabrication of the two series of thermo-responsive soft composites are presented in Fig. 2. As seen in this figure, the IPN of the final biocomposite hydrogel is comprised of two individual crosslinked networks: (i) PNIPAm crosslinked with MBA (reaction Scheme no. 1; network 1) and (ii) lignin–PVA crosslinked with GA (reaction Scheme no. 2; network 2), both of which are carried out under acidic conditions.

**Fig. 3:**
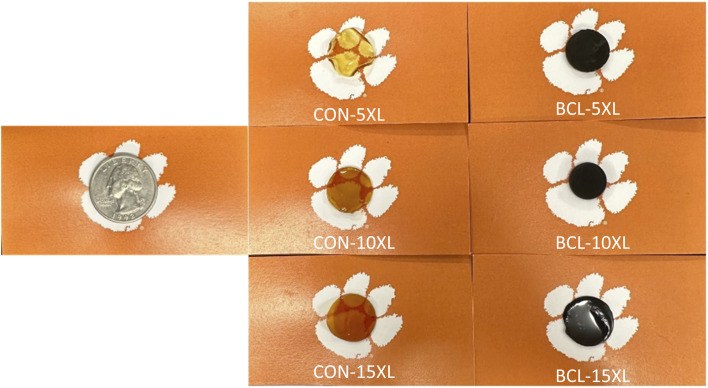
Images of the hydrated, free-standing PNIPAm–PVA control (CON) hydrogel membranes and lignin–PNIPAm–PVA biocomposite hydrogel membranes containing BioChoice lignin (BCL). In each column, the crosslinker concentration increases progressively from 5 to 15 mass%, moving from top to bottom. Note that the diameter of a United States quarter is approximately 25 mm.

Following the reaction schemes shown in Fig. 2, robust, free-standing membranes were successfully fabricated and are shown in Fig. 3. From top to bottom, the crosslinker content increases from 5 to 15 mass% of the respective crosslinked polymers. Each membrane shown was saturated in DI water for at least 24 hours and has an approximate thickness of 250 microns. As shown in Fig. 3, the orange color of the CON membranes intensifies with higher crosslinker content, resulting in reduced transparency. In contrast, the hydrogel membrane becomes completely opaque upon the lignin incorporation, irrespective of crosslinker content.

### 3.2. Equilibrium water uptake (EWU)

**Fig. 4:**
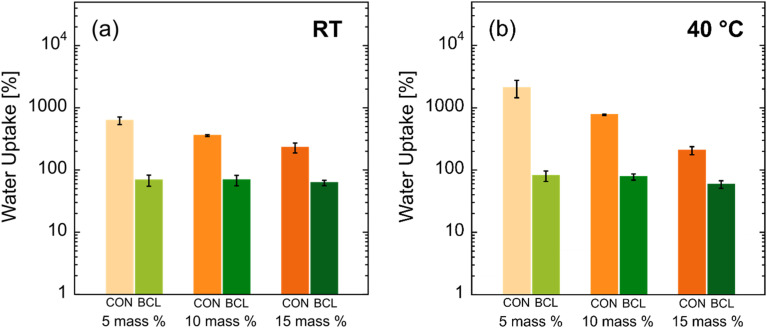
Equilibrium water uptake (EWU) for PNIPAm–PVA control (CON) hydrogel membranes and lignin–PNIPAm–PVA biocomposite hydrogel membranes (BCL) at (a) RT and (b) 40 °C. The error bars represent the standard deviation of the average EWU calculated from measurements on (at least) three separate membranes.

Following fabrication, the equilibrium water uptake (EWU) for each hydrogel was measured at room temperature (RT) and 40 °C. The results of this characterization are shown in Fig. 4. We note that a statistical significance analysis for these data is provided in Table S2 in the SI. As seen in Fig. 4, the introduction of BCL significantly reduces the EWU of the biocomposite hydrogels, both at RT and at 40 °C. This reduction is most apparent for CON-5XL and BCL-5XL hydrogels, where a reduction of over an order of magnitude in EWU is observed. This result is expected due to the more hydrophobic nature of lignin when compared to PVA. We attribute the larger error bars observed for CON-5XL hydrogels at room temperature and 40 °C to a likely increase in the heterogeneity of the IPN network, compared to hydrogels synthesized with 10 and 15 mass% crosslinker. At the crosslinker concentration used, only about 2% of the hydroxyl groups in the PVA were available for crosslinking, which likely contributed to greater network inhomogeneity. With regards to CON membranes, at both RT and 40 °C, the equilibrium water uptake of the hydrogels was seen to decrease with increasing crosslinker concentration. Again, this is expected, as higher concentrations of crosslinker should, in theory, result in a higher number of chemical crosslinks—*i.e.*, increased crosslink density or tighter network structure—and thus, a decrease in volume available for water molecules among the crosslinked polymer chains. For CON-5XL and CON-10XL hydrogels, the increase in temperature correlates to an increase in water uptake, up to two-fold for CON-10XL hydrogels. This result implies that when heated, the increase in water solubility of the PVA in the IPN exceeds the amount of water that is ‘forced’ out of the network due to the collapse of PNIPAm chains. This effect was negligible at the highest crosslinker concentration, where CON-15XL hydrogels did not show a measurable change in EWU between RT (≈230 ± 42%) and 40 °C (≈207 ± 30%). That is, at the highest crosslinker concentration, a balance was achieved between the swelling of the PVA network and the shrinking of the PNIPAm network due to the rise in temperature, where the more tightly constricted PVA network was not able to uptake as much water as its 5XL and 10XL counterparts.

In contrast, the impact of crosslinker concentration of biocomposite hydrogels containing BCL was negligible, where almost no change in EWU was observed among the three lignin-containing hydrogels. We posit that the lack of impact of lignin on the EWU of these hydrogels is due to the steric hindrance of the highly branched lignin chains, preventing efficient crosslinking of the PVA and PNIPAm in the IPN. In this case, it is the inherent hydrophobic nature of the lignin, and not the crosslink density, that controls the overall hydrophilicity (or EWU) of the biocomposite hydrogels, where increases in crosslinker concentration did not appear to result in more crosslinks between lignin chains.

### 3.3. Caffeine release kinetics and diffusivity

**Fig. 5:**
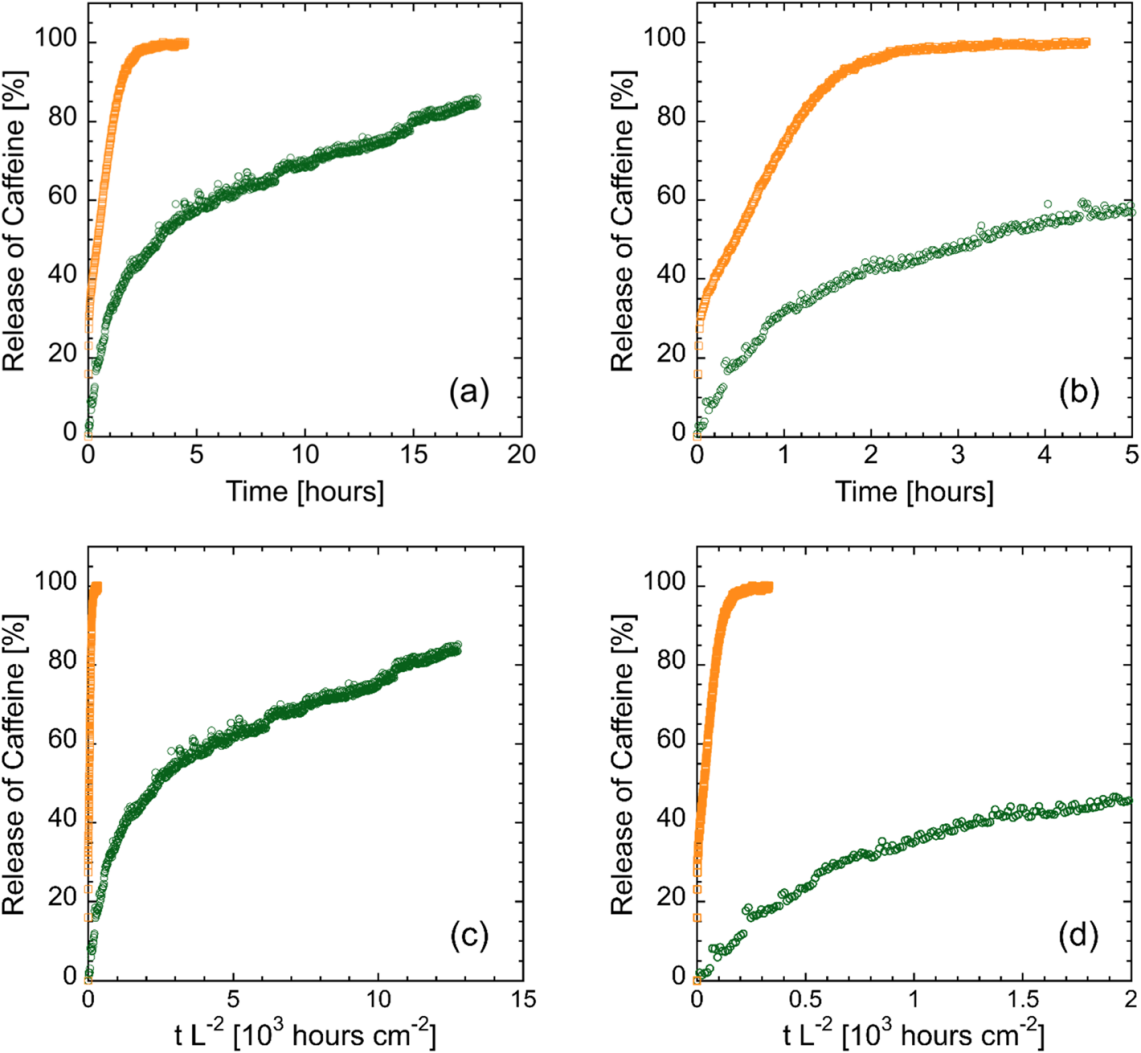
Caffeine release profiles for a PNIPAm–PVA control (CON, orange squares) hydrogel membrane and a lignin–PNIPAm–PVA biocomposite hydrogel membrane (BCL, green circles) at 15 mass% crosslinker and RT for (a) up to 18 hours, (b) the first hour, (c) the release profile normalized by thickness, centered for the BCL hydrogel profile, and (d) the release profile normalized by thickness, enlarged to capture the CON hydrogel profile.

To investigate the impact of lignin and crosslinker concentrations on drug release kinetics, the diffusion of caffeine—a water-soluble model drug—was characterized at room temperature (RT) and 40 °C. Fig. 5a shows representative caffeine release profiles for both a control (CON) hydrogel and a lignin-containing (BCL) hydrogel. Specifically, Fig. 5a and b show the full and early-time release profiles, respectively, for CON-15XL (open orange squares) and BCL-15XL (open green circles) hydrogels at RT. To ensure the difference in release kinetics are not a result of differences in membrane thickness across samples, the *x*-axis was also normalized by the square of each membrane's thickness (*L*^2^). This normalization reflects the fact that the characteristic diffusion time scale (*τ*_D_) is proportional to *L*^2^/*D*. The normalized release profiles are presented in Fig. 4c and d, showing the full and early-time data, respectively. After accounting for variations in membrane thickness, the faster release of caffeine from the CON-15XL hydrogel, relative to the BCL-15XL, becomes even more pronounced. Since the observed differences in release kinetics cannot be attributed to membrane thickness, the following discussion will focus on the unnormalized data shown in Fig. 5a and b.

As shown in Fig. 5a, caffeine release from the CON-15XL hydrogel was completed within the first four hours of the experiment. The quick, immediate release of all the loaded caffeine from the hydrogel is indicative of burst release,^13^ which again, is an undesired mechanism for most drug delivery. In contrast, over the same time, the BCL-15XL hydrogel released only ≈60% of the loaded caffeine. Similar results were found by Cheaburu-Yilmaz and coworkers,^23^ who observed that the release of Voriconazole was halved (from 76% to 38%) over a 6-hour period with the introduction of chitosan-grafted-PNIPAm into PVA hydrogels. Furthermore, the overall shape of the caffeine release profile for the BCL-15XL hydrogel is indicative of controlled, sustained drug release.^36^ This behavior may be attributed to the phenolic nature of both lignin and caffeine. It is well established that caffeine can interact strongly with phenolic groups through a combination of hydrogen bonding and hydrophobic interactions.^37^

The sustained release observed in the BCL hydrogels underscores the potential to harness these interactions to modulate the release kinetics of caffeine and other drug molecules, many of which share similar functional groups with caffeine. We would like to note that these potential molecular interactions that occur between caffeine and lignin in the lignin-containing membranes are inferred from the UV-vis data, as evidenced by the markedly reduced initial burst release and the significantly prolonged release kinetics compared to non-lignin-containing membranes. These effects extend well beyond what can be attributed solely to longer residence times associated with smaller pore sizes. Nonetheless, we acknowledge the limitations of this conclusion and recognize that fully elucidating the release mechanism will require additional molecular-level characterizations, such as Fourier transform infrared or Raman spectroscopy.

**Fig. 6:**
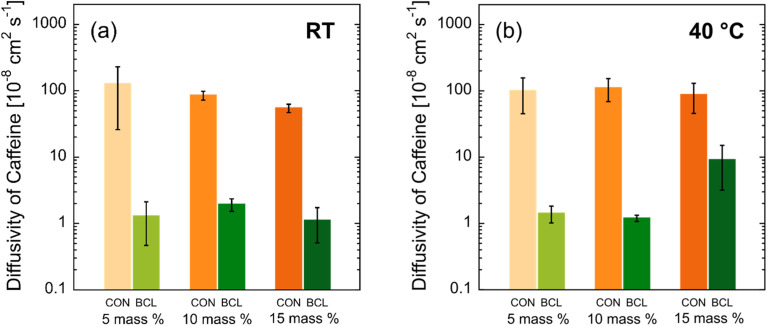
Caffeine diffusivity for PNIPAm–PVA control (CON) hydrogel membranes and lignin–PNIPAm–PVA biocomposite hydrogel membranes (BCL) at (a) RT and (b) 40 °C. The error bars represent the standard deviation of the average diffusivity calculated from measurements on (at least) three separate membranes.

In the early-time data presented in Fig. 5b, the slope of the release curve for the lignin-containing hydrogel is noticeably reduced, further highlighting the slower release of caffeine from these hydrogels. For each release profile, the early-time caffeine release data were regressed to eqn (1), where the effective diffusion coefficient, *D*_eff_, was the only adjustable parameter. The results of this analysis are shown in Fig. 6. We note that the mutual diffusion coefficient of caffeine in water is approximately 7 × 10^−6^ cm^2^ s^−1^.^38^ Similar to that for Fig. 4, a statistical significance analysis for these data is provided in Table S3 in the SI. For CON hydrogels at RT (Fig. 6a), there was no measurable difference in the diffusion coefficient of caffeine with increasing crosslinker concentration, with the effective caffeine diffusivity calculated to be approximately 1 × 10^−6^ cm^2^ s^−1^. This may be a result of the small size of the caffeine molecule, with dimensions less than 1 nm in size.^39^ Although increasing the crosslinker concentration increases the crosslink density (and thus reduces the mesh size), it does not do so to a degree that decreases the average mesh size to that of the caffeine molecule. As a result, caffeine can diffuse out of the IPN with minimal hindrance from the crosslinked network structure.

As seen from Fig. 6a, the incorporation of lignin results in approximately a two-order-of-magnitude decrease in caffeine diffusivity (∼1 × 10^−6^ cm^2^ s^−1^*vs.* ∼1 × 10^−8^ cm^2^ s^−1^). Similar to the behavior observed for CON hydrogels, the diffusion coefficient of caffeine was seen to be independent of crosslinker concentration, falling within the range of 1–2 × 10^−8^ cm^2^ s^−1^. This reduction in diffusivity may result from a combination of: (1) strong ionic interactions between caffeine and lignin, due to lignin's high phenolic content and the presence of hydroxyl and carboxylic acid groups, and (2) a reduced average mesh size in BCL hydrogels, likely caused by both lignin crosslinking and increased physical entanglements stemming from lignin's highly branched polymer structure. In contrast, CON hydrogels, where lignin is replaced by linear (uncrosslinked) PVA, lack such native physical crosslinks. Consequently, under equivalent chemical crosslinker concentrations, PVA networks form fewer total crosslinks than lignin–PVA networks, resulting in a looser network and faster caffeine diffusion. Zhu and collaborators reported similar findings in PVA–graphene oxide (GO) hydrogels for wound dressings, where the incorporation of GO—which is known to introduce additional intermolecular crosslinks—led to a reduced release rate, which is similar to the effect of lignin observed in this study.^40^

The most pronounced increase in caffeine release between RT and 40 °C occurred in both BCL and CON membranes containing 15 mass% crosslinker, while membranes with lower crosslinker concentrations showed minimal temperature-dependent changes. This significant increase in diffusivity at 40 °C is unexpected, as higher crosslinker concentrations typically yield denser networks that resist thermal expansion. We propose that, in the 15 mass% crosslinked hydrogels, thermoresponsive behavior was amplified, accelerating caffeine release upon heating. The absence of an increase in EWU for CON-15XL membranes suggests a delicate balance between water absorption by PVA and thermally induced water expulsion from PNIPAm. In this system, the PNIPAm network likely underwent localized shrinkage, generating a physical force that expelled caffeine more rapidly. Additionally, the higher crosslinker concentration may have altered microphase separation or reduced polymer compatibility, creating channels that facilitated diffusion. Together, these effects led to an order-of-magnitude increase in caffeine diffusivity in the 15 mass% crosslinked hydrogels at elevated temperature. Finally, the large standard deviation observed for CON-5XL membranes at both RT and 40 °C aligns with the expectation that a low crosslinker concentration produces a loosely structured, heterogeneous network, resulting in variability across samples.

### 3.4. Scanning electron microscopy characterization of hydrated network

**Fig. 7:**
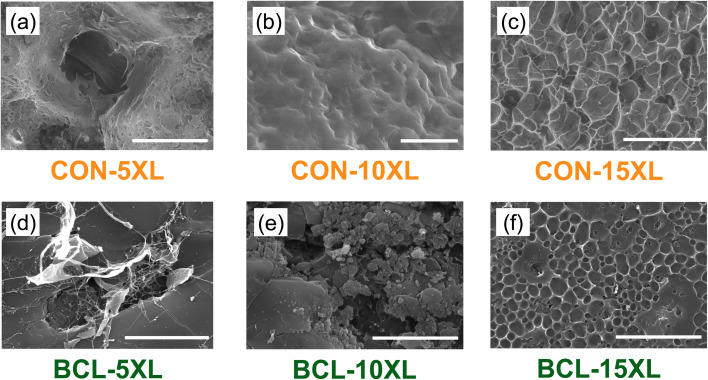
SEM images of CON hydrogels at (a) 5 mass%, (b) 10 mass%, and (c) 15 mass% crosslinker, and BCL hydrogels at (d) 5 mass%, (e) 10 mass%, and (f) 15 mass% crosslinker. All hydrogels were hydrated in water at room temperature. Note that the scale bar in all the images is 100 μm.

To gain insight into the caffeine release behavior described previously, the network structures of the hydrated hydrogels at both room temperature (RT) and 40 °C were examined using scanning electron microscopy (SEM). The resulting images are shown in Fig. 7, with CON and BCL membranes presented in the top and bottom rows, respectively, and crosslinker concentration increasing from left to right. Each image includes a 100 μm scale bar.

Beginning with the CON hydrogels (top row, Fig. 7), minimal or no visible crosslinked network structure is evident for CON-5XL and CON-10XL at this scale. The heterogeneous appearance of the CON-5XL image (Fig. 7a) may help explain the high variability in measured caffeine diffusivity for this sample. In contrast, a distinct network structure emerges in CON-15XL, where pores on the order of tens of microns are visible. This more defined and uniform pore structure may contribute to the reduced caffeine diffusivity observed when increasing crosslinker content from 10 to 15 mass%, decreasing from approximately ∼9 × 10^−7^ cm^2^ s^−1^ to ∼6 × 10^−7^ cm^2^ s^−1^.

In the BCL hydrogels (bottom row, Fig. 7), a different network morphology is observed. SEM images of BCL-5XL and BCL-10XL reveal no clear polymer network. Instead, large lignin aggregates dominate the microstructure. This lack of network structure mirrors that seen in CON hydrogels with equivalent crosslinker content. As with the CON series, increasing the crosslinker content to 15 mass% in BCL-15XL results in a more defined network, with pores roughly 10 μm in size. However, several areas in the image show large lignin “islands” that disrupt the formation of these pores. Interestingly, the pore size distribution in BCL-15XL appears more uniform than in CON-15XL, and many pores seem to be filled with lignin. These observations suggest that, at RT, the presence and structure of the hydrated network have less influence on caffeine release in BCL hydrogels than the lignin content itself. Since all BCL samples contain the same amount of lignin and exhibit similar caffeine diffusivity, it is likely that direct interactions between caffeine and lignin^41^ primarily govern the more controlled release seen in these systems.

**Fig. 8:**
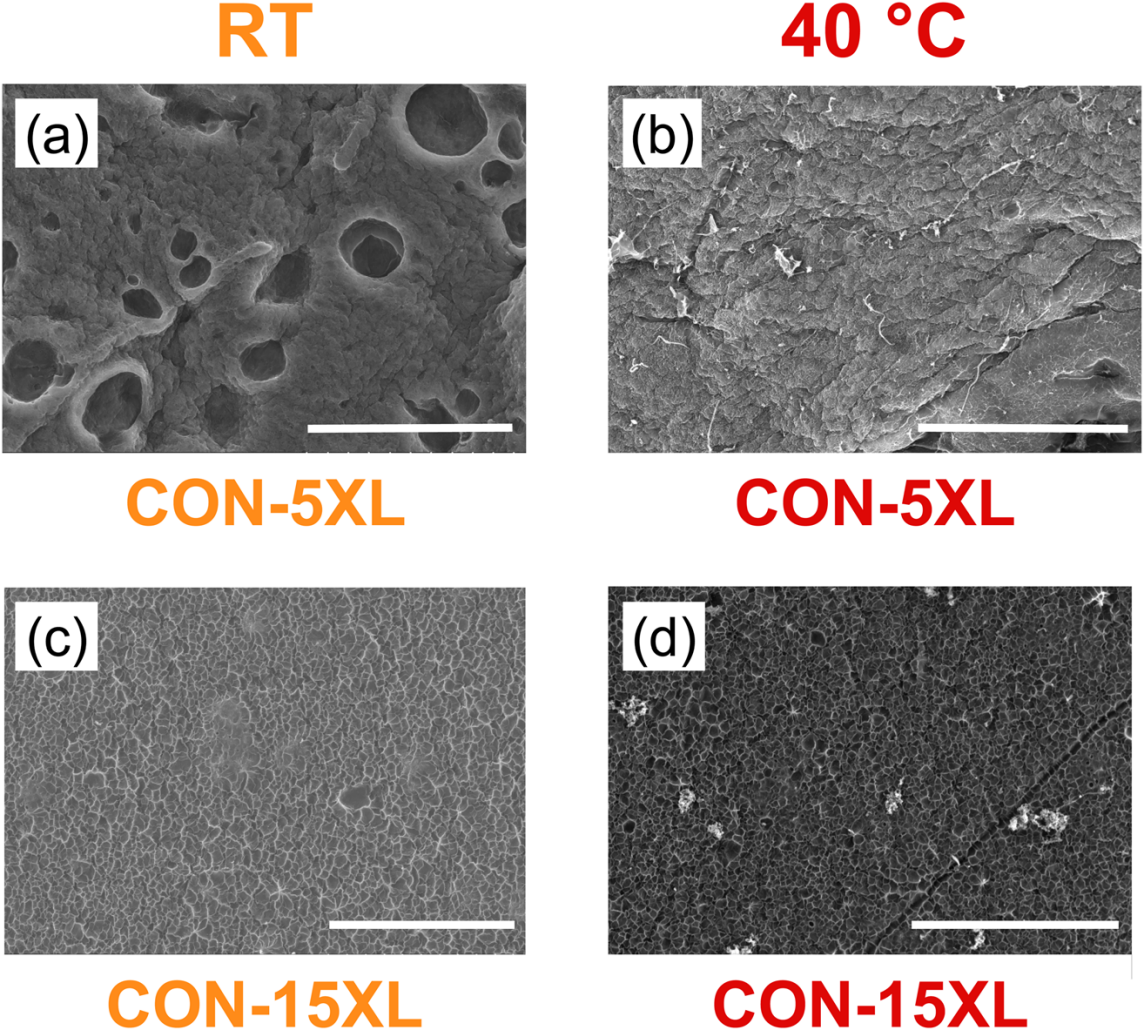
SEM images of CON hydrogels with 5 mass% crosslinker at (a) RT and (b) 40 °C and with 15 mass% crosslinker at (c) RT and (d) 40 °C. Note that the scale bar in all the images is 500 μm.

The effect of temperature on the hydrated network structure was examined by lyophilizing hydrogels that were equilibrated in water at 40 °C, a temperature well above the VPPT of PNIPAm.^42^Fig. 8 presents the SEM images of CON hydrogels prepared with 5 and 15 mass% crosslinker, both at RT and at 40 °C. The scale bar in each image represents 500 μm. As shown in Fig. 8a, the CON-5XL hydrogel exhibits large pores—hundreds of microns in size—visible on both the surface and throughout the thickness of the sample, consistent with the higher magnification image in Fig. 7a. When heated to 40 °C (Fig. 8b), these pores disappear completely, reflecting the thermoresponsive contraction of the PNIPAm network.^43^ Although the pore structure collapses, a faint crosslinked network remains visible, albeit difficult to resolve at this scale. In contrast, the CON-15XL hydrogels (Fig. 8c and d) show little to no change in structure upon heating. This lack of response is likely due to the increased crosslink density, which restricts chain mobility and suppresses the network's ability to undergo thermal contraction. This similarity in hydrated network structure may help to explain the similar caffeine diffusivity for these two hydrogels, where there was no measurable difference is calculated diffusivities (see dark orange bar in Fig. 6a and b).

**Fig. 9:**
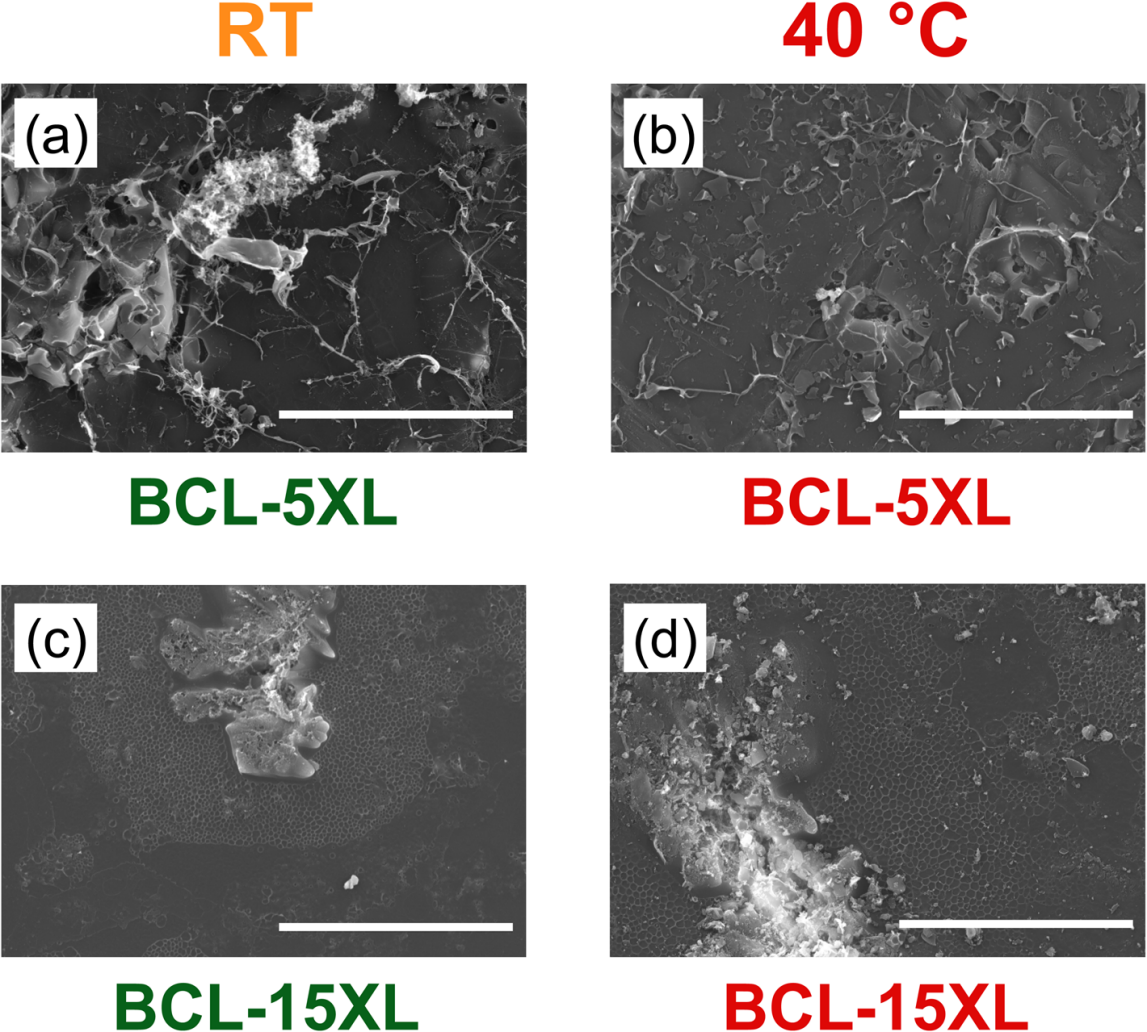
SEM images of BCL hydrogels with 5 mass% crosslinker at (a) RT and (b) 40 °C and with 15 mass% crosslinker at (c) RT and (d) 40 °C. Note that the scale bar in all the images is 500 μm.

Fig. 9 presents SEM images of BCL hydrogels prepared with 5 and 15 mass% crosslinker, imaged at both RT and 40 °C. Each image includes a 500 μm scale bar. Notably, for BCL-5XL, no discernible hydrated network is visible at this scale, regardless of temperature—an observation consistent with the higher magnification images previously shown in Fig. 7d. In contrast, the BCL-15XL hydrogels exhibit a distinct porous network that, while relatively uniform in texture, appears in isolated “islands”, interspersed with regions lacking noticeable structure.

Upon heating to 40 °C, the BCL-15XL hydrogels reveal a more continuous and extensive porous network across the SEM field of view. This structural change may contribute to the nearly tenfold increase in caffeine diffusivity observed at elevated temperatures. The expanded porous morphology suggests that the thermal collapse of the PNIPAm network influences molecular transport—an effect that appears unique to the lignin-containing system. Interestingly, this enhanced diffusivity occurs despite BCL-15XL exhibiting similar EWU values at both RT and 40 °C (Fig. 4a*vs.*4b). This finding suggests a decoupling between bulk hydrophilicity and drug diffusivity, further underscoring the complex and distinctive behavior of these lignin-based hydrogels.

## 4. Conclusions

In this study, we examined the effects of lignin incorporation and crosslinker content on the water uptake and caffeine diffusivity of lignin–PNIPAm–PVA hydrogels. For PNIPAm–PVA hydrogels without lignin, a decrease in EWU with increasing crosslinker concentration confirmed the formation of a denser, more tightly crosslinked network. However, this trend was not observed in lignin-containing hydrogels, where increased crosslinker content did not lead to a corresponding decrease in EWU. This suggests that the replacement of PVA with lignin disrupts the ability to modulate water affinity through crosslinking, likely due to differences in chemical structure and network integration.

Across all hydrogel compositions, the inclusion of lignin resulted in a significant reduction in caffeine diffusivity, by up to two orders of magnitude, compared to lignin-free controls. Importantly, lignin-containing hydrogels exhibited no “burst release” in drug release kinetics, a behavior clearly present in CON hydrogels without lignin. Among lignin-containing samples, BCL-15XL hydrogels at 40 °C demonstrated a modest increase in caffeine diffusivity relative to both their RT counterparts and lower-crosslinked BCL hydrogels at 40 °C. Despite this change in transport behavior, SEM analysis revealed minimal differences in the hydrated porous structure of BCL-15XL at RT *versus* 40 °C. Overall, caffeine diffusivity and EWU remained largely unaffected by crosslinker concentration or temperature within each hydrogel type, suggesting a decoupling of network structure and transport properties in the presence of lignin.

These findings point to a consistent disruption of mesh-like network formation in lignin-containing hydrogels, where crosslinker concentration does not significantly influence water uptake or drug diffusivity. This consistency offers a valuable design advantage: the potential to tailor other hydrogel properties while maintaining controlled drug release rates. We note that future work will expand to include larger, macromolecules such as Dextran, as their incorporation (and release) will help establish the broader applicability of this platform for a wide range of therapeutic agents. Even with this, we believe lignin–PNIPAm–PVA hydrogels represent a promising, renewable alternative for sustainable, cost-effective, and high-performance drug delivery platforms.

## Author contributions

Missoury Wolff: formal analysis, investigation, writing – original draft and review & editing, visualization. Eric M. Davis: conceptualization, validation, writing – review & editing, visualization, supervision.

## Conflicts of interest

The authors declare no competing financial interest.

## Supplementary Material

RA-015-D5RA05933J-s001

## Data Availability

The data that support the findings of this study are available from the corresponding author upon reasonable request. Supplementary information: crosslinking capacity calculations and statistical analysis of the experimental data. See DOI: https://doi.org/10.1039/d5ra05933j.
