# Supplementary material for: Smart biocomposite hydrogels in action: unraveling the roles of lignin, temperature, and crosslinker on drug release
Source: RSC Adv. 2025 Nov 7;15(51):43345–56. doi: 10.1039/d5ra05933j (PMC12593194; doi:10.1039/d5ra05933j)
Supplement: RA-015-D5RA05933J-s001 [file RA-015-D5RA05933J-s001.pdf]

Supplementary Information (SI) for RSC Advances.
This journal is © The Royal Society of Chemistry 2025

Electronic Supporting Information (ESI)

for

Smart Biocomposite Hydrogels in Action: Unraveling the Roles of Lignin, Temperature, and Crosslinker on Drug Release

*Missoury Wolff and Eric M. Davis**

Department of Chemical and Biomolecular Engineering, Clemson University, Clemson, South Carolina 29634

*To whom correspondence should be addressed; Email: ericd@clemson.edu

Maximum (Theoretical) Crosslinking Capacities for Each Chemical Compositions

As crosslinking occurs within each network, a mesh is formed at the junction of the crosslinker and polymer functional groups. To assess the relative capacity of each crosslinker concentration to create the network structure, the percentage of crosslinked functional groups was calculated and presented in **Table S1**. Note that the percentages were calculated with the assumption that each crosslinker can successfully crosslink two functional groups. Therefore, the value presented here represents the maximum fraction of groups crosslinked within each network.

Table S1. Percentages of crosslinked groups of interest that correspond to various crosslinker concentrations. Note, negative quantities in millimole per gram constitute groups consumed by crosslinking.

		**Percent of Crosslinked [–OH] Groups**					
		Control Hydrogel			Contains Lignin		
**Chemical**	**[–OH] groups**	5 mass % XL	10 mass % XL	15 mass % XL	5 mass % XL	10 mass % XL	15 mass % XL
	[mmol g^{-1}]						
Lignin	**+ 6.45**						
PVA	**+22.70**						
GA *Soln*	**-19.98**	**4.43%**	**9.35%**	**14.9%**	**2.32%**	**4.89%**	**7.76%**
		**Percent of Crosslinked Isopropyl/Amide Groups**					
**Chemical**	**Isopropyl & amide groups**	5 mass % XL		10 mass % XL		15 mass % XL	
	[mmol g^{-1}]						
PNIPAm	**+8.84**						
MBA	**-12.97**	**7.72%**		**16.3%**		**25.9%**	

Statistical Analysis

To improve the rigor of our discussion of the experimental data, unpaired t tests were performed on the equilibrium water uptake (EWU) and caffeine diffusivity data to determine if there was a statistical significance in the measured difference in these values between the temperature, lignin incorporation, and crosslinker content. Any t test that yielded a two-tailed p

value less than or equal to 0.05 (or 5%) was deemed statistically significant. Analysis was performed with GraphPad (graphpad.com). The results of this analysis for the datasets are summarized in **Tables S2** and **S3**.

Table S2. Summary of statistical significance of equilibrium water uptake (EWU) values of the membranes investigated in this study. Here, the values tabulated are the two-tailed p values, where ✓'s indicate statistical significance between samples and ×'s indicate a lack of statistical significance.

**EWU**	**BCL at RT to CON at RT**	**BCL at 40 °C to CON at 40 °C**	**BCL at RT to BCL at 40 °C**	**CON at RT to CON at 40 °C**
**5XL**	<0.0001 ✓	0.0058 ✓	0.3242 ×	0.0177 ✓
**10XL**	<0.0001 ✓	<0.0001 ✓	0.3652 ×	<0.0001 ✓
**15XL**	0.0005 ✓	0.0033 ✓	0.5790 ×	0.5608 ×
**RT**	**BCL-5XL**		**BCL-15XL**	
**BCL-5XL**	–		0.4309 ×	
**BCL-10XL**	0.9849 ×		0.3955 ×	
**40 °C**	**BCL-5XL**		**BCL-15XL**	
**BCL-5XL**	–		0.0956 ×	
**BCL-10XL**	0.7743 ×		0.0536 ×	

Table S3. Summary of statistical significance of caffeine diffusivity values of the membranes investigated in this study. Here, the values tabulated are the two-tailed p values, where ✓'s indicate statistical significance between samples and ×'s indicate a lack of statistical significance.

Diffusivity	BCL at RT to CON at RT	BCL at 40 °C to CON at 40 °C	BCL at RT to BCL at 40 °C	CON at RT to CON at 40 °C
**5XL**	0.0505 ×	0.0150 ✓	0.2004 ×	0.7078 ×
**10XL**	0.0004 ✓	0.0107 ✓	0.0407 ✓	0.3734 ✓
**15XL**	0.0003 ✓	0.0022 ✓	0.0815 ×	0.0470 ✓
**RT**	**BCL-5XL**		**BCL-15XL**	
**BCL-5XL**	–		0.7756 ×	
**BCL-10XL**	0.2703 ×		0.1239 ×	
**40 °C**	**BCL-5XL**		**BCL-15XL**	
**BCL-5XL**	–		0.1029 ×	
**BCL-10XL**	0.2261 ×		0.0827 ×	
